# Adaptation to sustained nitrogen starvation by *Escherichia coli* requires the eukaryote-like serine/threonine kinase YeaG

**DOI:** 10.1038/srep17524

**Published:** 2015-12-01

**Authors:** Rita Figueira, Daniel R. Brown, Delfim Ferreira, Matthew J. G. Eldridge, Lynn Burchell, Zhensheng Pan, Sophie Helaine, Sivaramesh Wigneshweraraj

**Affiliations:** 1 MRC Centre for Molecular Microbiology and Infection, Imperial College London, UK

## Abstract

The *Escherichia coli* eukaryote-like serine/threonine kinase, encoded by *yeaG*, is expressed in response to diverse stresses, including nitrogen (N) starvation. A role for *yeaG* in bacterial stress response is unknown. Here we reveal for the first time that wild-type *E. coli* displays metabolic heterogeneity following sustained periods of N starvation, with the metabolically active population displaying compromised viability. In contrast, such heterogeneity in metabolic activity is not observed in an *E. coli* ∆*yeaG* mutant, which continues to exist as a single and metabolically active population and thus displays an overall compromised ability to survive sustained periods of N starvation. The mechanism by which *yeaG* acts, involves the transcriptional repression of two toxin/antitoxin modules, *mqsR/mqsA* and *dinJ*/*yafQ*. This, consequently, has a positive effect on the expression of *rpoS*, the master regulator of the general bacterial stress response. Overall, results indicate that *yeaG* is required to fully execute the *rpoS*-dependent gene expression program to allow *E. coli* to adapt to sustained N starvation and unravels a novel facet to the regulatory basis that underpins adaptive response to N stress.

Continuous bacterial growth is often limited by the availability of key nutrients. Since numerous cellular macromolecules contain nitrogen (N), including proteins, nucleic acids and cell wall components, *Escherichia coli* and other members of the *Enterobacteriaceae* family immediately cease growth upon sensing N starvation and simultaneously initiate two major adaptive responses: the nitrogen regulation (Ntr) stress response and the stringent response^1^. The Ntr response primarily confers the ability to scavenge for alternative N sources, through the transcriptional activation of genes that encode transport systems and catabolic or biosynthetic operons by the global transcriptional regulator NtrC^2^. The stringent response is mediated by the alarmone guanosine tetraphosphate (ppGpp), which primarily affects the transcriptional program of the bacterial cell: ppGpp influences the competition between different RNA polymerase-associated promoter-specificity sigma (σ) factors, so that different stress-related σ factors (notably σ^38^ and σ^54^) bind to the RNA polymerase at the expense of the housekeeping σ factor, σ^70^ 3. Collectively, this results in the adjustment of cellular metabolism to divert resources away from expression of genes required for normal growth to ones that ensure survival until nutrient conditions improve. We recently demonstrated that NtrC activates transcription of *relA*, the major gene responsible for the synthesis of ppGpp, in N-starved *E. coli*^4^. Thus, NtrC, by coupling the Ntr and stringent responses in N-starved *E. coli*, facilitates efficient and rapid adaptation to N starvation.

Genome-wide binding analysis in N-starved *E. coli* revealed that NtrC and RNA polymerase bind the promoter region of the *yeaGH* operon, thus implying that the products of *yeaG* and *yeaH* could have a role in the adaptive response of *E. coli* to N starvation^4^. Previous global transcriptome analyses have revealed that the expression of *yeaG* and *yeaH* are highly upregulated in *E. coli* in response to diverse stresses, including low pH, hyperosmotic conditions, entry into stationary phase and sulphur limitation^5^,^6^. Moreover, in *Salmonella* Typhimurium these genes are also required in the response against the antimicrobial peptide Polymyxin B^7^. Therefore, it is possible that *yeaG* and *yeaH* may have an important role in how bacteria adapt to diverse stress conditions. Consistent with this view, the *yeaGH* operon is highly conserved across bacterial species, especially in *Enterobacteria* (Figure S1A). Whereas *yeaH* encodes an uncharacterized protein that displays very little sequence or structural similarity to any known proteins described to date, the product of *yeaG*, YeaG, is a 75 kDa protein containing a carboxyl terminal domain that shares amino acid sequence homology to eukaryote-like serine/threonine kinase (eSTK) (Figure S1B). eSTKs have emerged as critical components of bacterial signal transduction systems and are central to important and diverse cellular functions, including bacterial stress response^8^,^9^. Consistent with their different biological roles, many eSTKs are modular and display a high diversity in domain organization^10^. Therefore, the kinase domain of many eSTKs is associated with additional domains, which commonly mediate signal perception through ligand binding or protein-protein interactions^11^. A small number of eSTKs are also associated with enzymatic domains (pectinases, esterases, phosphatases, etc.)^11^. Intriguingly, YeaG contains an amino-terminal ATPase Associated with diverse cellular Activities (AAA+) domain (Figure S1C). Typically, AAA+ domain-containing proteins are oligomeric mechanochemical enzymes that transform chemical energy derived from ATP binding and hydrolysis into mechanical force to induce conformational changes in their substrates^12^. Therefore, it seems that YeaG is an atypical, yet highly conserved, eSTK. Although a previous study demonstrated that recombinant YeaG has protein kinase activity *in vitro*^13^, surprisingly the biological role of YeaG in bacterial stress responses remains elusive. In this study, we have investigated the role of *E. coli* YeaG in the adaptive response to N starvation.

## Results

### *yeaG* has a role in the adaptive response to sustained N starvation

**Figure 1:**
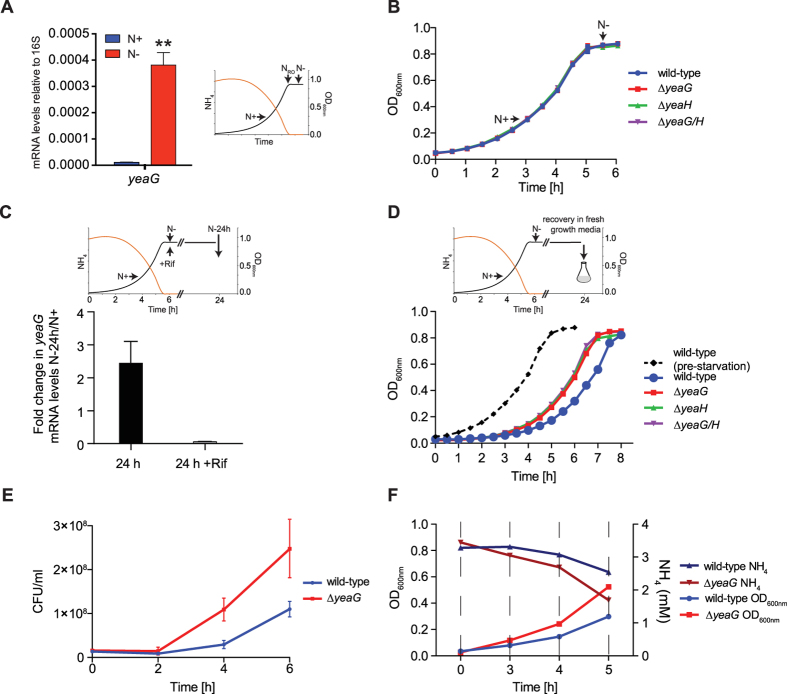
*yeaG* has a role in the adaptive response to sustained N starvation. (**A**) *yeaG* is upregulated in response to N starvation. Levels of *yeaG* expression in wild-type *E. coli* were calculated as relative mRNA amounts compared to the 16S control under N-replete (N+) and N-starved (N-) conditions. The *insert* is a schematic graph showing that *E. coli* growth arrest corresponds to N run out (N_RO_) in the media (orange line) and indicates the time points (N+ and N-) at which cells were sampled for total RNA extraction and analysis. (**B**) ‘Pre-starvation’ growth of ∆*yeaG*, ∆*yeaH* and ∆*yeaGH* mutant strains is similar to that of the wild-type strain. The growth curves were obtained by determining the OD_600nm_ readings at half-hourly time points. (**C**) Transcription of *yeaG* occurs during sustained (24 h) N starvation. The level of *yeaG* expression in wild-type *E. coli* is expressed as fold change in mRNA levels between N+ and N-24 h starved conditions. In the presence of rifampicin, an inhibitor of RNA synthesis, no *yeaG* mRNA was detected in the N-24 h sample, which indicates that the *yeaG* mRNA expression observed in the absence of rifampicin is due to transcription of *yeaG* over the 24 h period of sustained N starvation. The *insert* is a schematic representation of the experiment. (**D**) ∆*yeaG,* ∆*yeaH* and ∆*yeaGH* mutant strains display shorter lag phase during ‘recovery’ growth following 24 h of N starvation compared to a wild-type strain. The ‘recovery’ growth curves were obtained by determining the OD_600nm_ readings at half-hourly time points. The dotted black line indicates the ‘pre-starvation’ growth curve for the wild-type strain. The *insert* is a schematic representation of the experiment. (**E**) Measurement of ‘recovery’ growth of wild-type and ∆*yeaG* mutant strain by colony-forming unit (CFU) count at 0 h, 2 h, 4 h and 6 h. (**F)** The rate of ammonium consumption by the wild-type and ∆*yeaG* mutant strain is directly proportional to their respective ‘recovery’ growth characteristics. Error bars on all growth curves and graph represent sd (*n* = 3). Statistical analyses were performed by one-way ANOVA (**P < 0.01).

**Figure 2:**
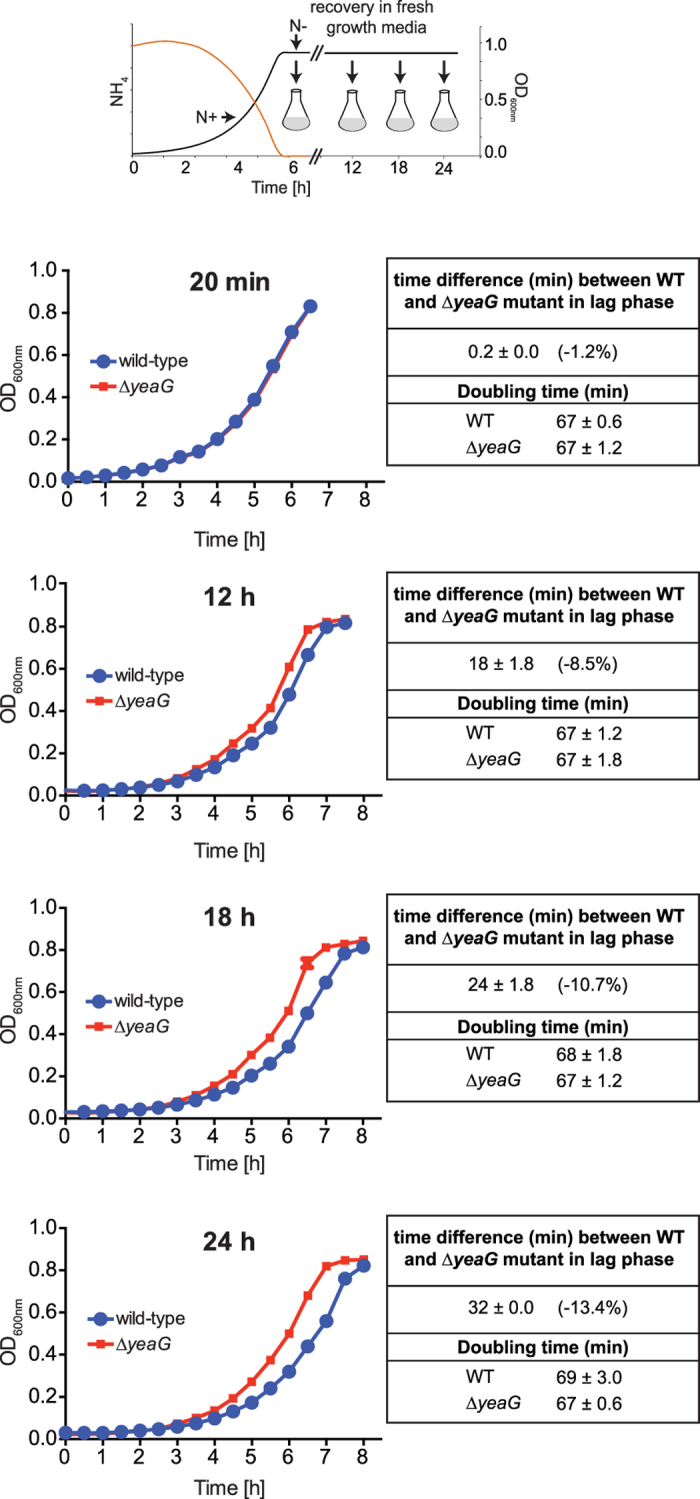
The difference in the length of the lag phase between the wild-type and ∆*yeaG* strain during ‘recovery’ growth is directly proportional to the length of time spent under N starvation. The ‘recovery’ growth curves of wild-type and ∆*yeaG* mutant strain were obtained by determining the OD_600nm_ readings at half-hourly time points. The *insert* is a schematic representation of the experiment. The table summarizes growth parameters of wild-type and ∆*yeaG* mutant strain in ‘recovery’ growth following 20 min, 12 h, 18 h and 24 h in N starvation. Lag phase defined as the period up to and including OD_600_ = 0.1. The number in brackets indicates the percentage decrease in the length of the lag phase of the ∆*yeaG* mutant strain relative to the lag phase of the wild-type strain. The doubling time was determined from the slope of logarithmic growth function.

Previously we reported that NtrC and RNA polymerase bind to the promoter region of the *yeaGH* operon in N-starved *E. coli*^4^. To establish whether these binding events lead to transcription of the *yeaGH* operon we measured levels of *yeaG* mRNA by quantitative real-time PCR. Bacteria were grown in batch cultures in minimal media that was supplemented with a limiting amount (3 mM) of ammonium as the sole source of N. Under these conditions, we previously showed that bacterial growth arrest directly coincides with the acquisition of a *bona fide* N starved state (indicated as N- in the schematic in Fig. 1A where OD_600_ is ~0.85 and [NH_4_Cl] is <0.000625 mM) that occurs 20 min after ammonium has run out in the media (indicated as N_RO_ in the schematic in Fig. 1A)^4^. As expected, *yeaG* mRNA levels in N-starved *E. coli* were 36-fold (±2.3 SD) higher than in bacteria from nitrogen-replete conditions (indicated as N+ in the schematic in Fig. 1A where OD_600_ is ~0.3 and [NH_4_Cl] is ~2.5 mM) (Fig. 1A). Having established that *yeaG* is expressed in N-starved *E. coli* shortly after sensing N starvation, we next determined the growth characteristics of a ∆*yeaG* mutant *E. coli* strain in minimal media containing limiting amount (3 mM) of ammonium as the sole N source. Under these conditions (henceforth referred to as ‘pre-starvation’ growth) no growth difference was detected between the ∆*yeaG* mutant and wild-type *E. coli* and both strains ceased growth when ammonium ran out in the media (Fig. 1B). Even though the expression of *yeaG* occurs shortly after the cells sense N starvation (Fig. 1A), it appears to be continuously expressed for up to 24 h into N starvation (Fig. 1C), we considered whether *yeaG* could have a role in adaptation to sustained (here defined as 24 h, unless otherwise indicated) N starvation. To investigate this, wild-type and ∆*yeaG* mutant bacteria were subjected to 24 h of N starvation before being sub-cultured into fresh media and their growth monitored (henceforth referred to as ‘recovery’ growth). As shown in Fig. 1D, the wild-type strain displayed a considerably increased lag phase (by 2.3 h) during ‘recovery’ growth compared to ‘pre-starvation’ growth, which is characteristic of bacterial adaptation to the new growth environment. In marked contrast to the wild-type strain, the ∆*yeaG* mutant strain displayed a much shorter lag phase (by 32 min or 13% less than the length of wild-type lag phase) during ‘recovery’ growth (Fig. 1D and Table S1). However, once in the exponential phase of ‘recovery’ growth, the doubling times of both strains were comparable (Fig. 1D and Table S1). Similar results were obtained with *E. coli* strains lacking *yeaH* or both *yeaG* and *yeaH* genes, indicating that *yeaG* and *yeaH* are functionally linked (Fig. 1D and Table S1). Control experiments in which we either measured the number of viable cells at different times during ‘recovery’ growth by plate count of colony forming units (CFU) (Fig. 1E) or monitored the rate of ammonium consumption during ‘recovery’ growth (Fig. 1F) independently validated the shorter lag phase of the ∆*yeaG* mutant compared to the wild-type strain. Importantly, the difference in the length of the lag phase between the wild-type and ∆*yeaG* strain during ‘recovery’ growth was directly proportional to the length of time spent under N starvation conditions (Fig. 2), which further emphasizes the role for *yeaG* in sustained N starvation, even though the expression of *yeaG* occurs shortly after the cells experience N starvation.

**Figure 3:**
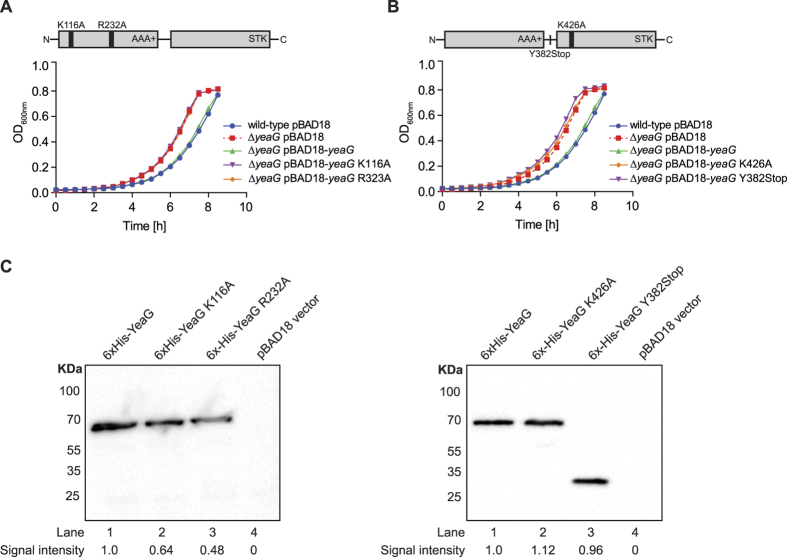
The catalytic activities of the AAA+ and eSTK domains of YeaG are required for its role in adaptation to sustained N starvation in *E. coli*. (**A,B**) Growth curves show complementation of the phenotype of the ∆*yeaG* mutant strain with plasmid-borne *yeaG* (pBAD18-*yeaG*), but not with mutant variants of *yeaG* containing catalytically deleterious mutations in the AAA+ domain (in A. pBAD18-*yeaG* K116A; pBAD18-*yeaG* R232A) or eSTK domain (in B. pBAD18-*yeaG* K426A; pBAD18-*yeaG* Y382Stop). Error bars on all growth curves represent sd (*n* = 3). Statistical analyses were performed by one-way ANOVA (**P < 0.01). (**C**) Protein levels of wild-type and mutant variants of YeaG assessed by anti-His immunoblotting.

We were able to revert the growth characteristics of the ∆*yeaG* mutant strain to that of the wild-type strain by complementing with inducible plasmid-borne *yeaG* (∆*yeaG* pBAD-*yeaG*), thereby demonstrating that the altered growth of the mutant strain is not due to any undesired effects caused by the deletion of the *yeaG* gene (Fig. 3A,B). To determine if the catalytic activity of the AAA+ and/or STK domains of YeaG are required for function, we constructed mutant variants of YeaG based on amino acid sequence alignments with AAA+ and STK domains of well-characterized proteins (Figure S1B and Figure S1C). These mutants contained catalytically deleterious single amino acid substitutions either in the AAA+ (pBAD18-*yeaG* K116A and pBAD18-*yeaG* R232A) or STK (pBAD18-*yeaG* K426A) domain. We also generated a truncated version of YeaG by introducing a stop codon at amino acid position Y382 (pBAD18-*yeaG* Y382stop), which results in only the AAA+ domain being expressed. As shown in Fig. 3A–C, even though the mutant variants were produced at 64% (*yeaG* K116A), 48% (*yeaG* R232A), 100% (*yeaG* K426A) and 96% (*yeaG* Y382stop) of wild-type levels, none of them were able to complement the phenotype of the ∆*yeaG* mutant strain under our experimental conditions. Overall, the results in Figs 1, 2, 3 clearly demonstrate that *yeaG* (and *yeaH*) has a role in the adaptation of *E. coli* to sustained N starvation and that the catalytic activities of both, the AAA+ and STK domains of YeaG, are required in this process.

### ∆*yeaG* mutant strain displays increased metabolic activity than the wild-type strain following sustained N starvation

**Figure 4:**
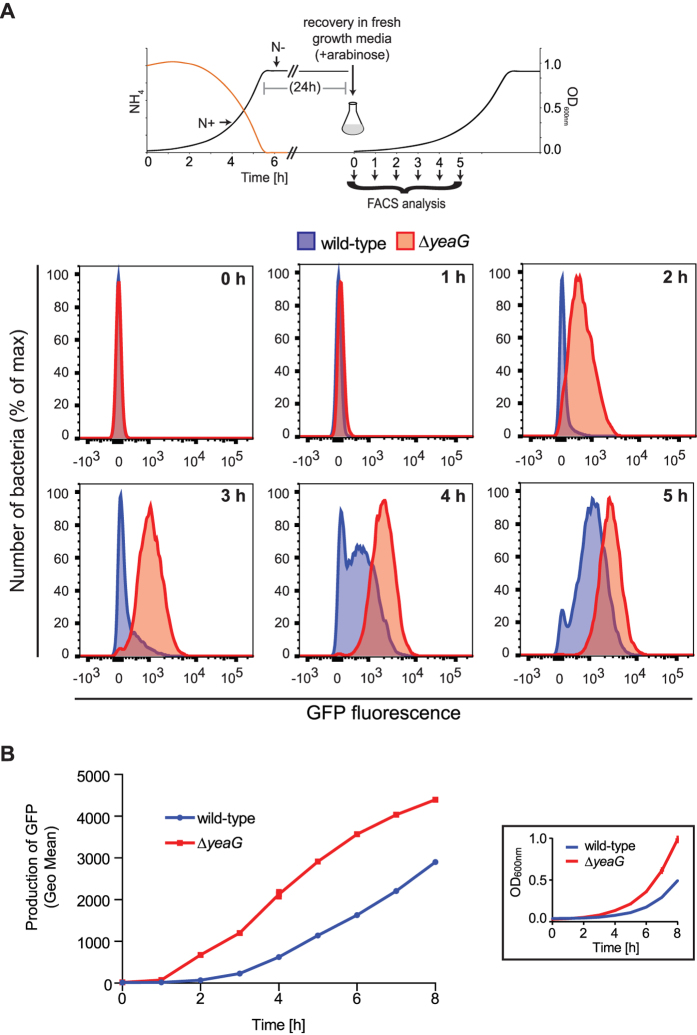
∆*yeaG* mutant strain displays increased metabolic activity than the wild-type strain following sustained N starvation. (**A**) Representative histograms of GFP fluorescence in wild-type (blue) and ∆*yeaG* mutant strain (red) populations at selected time points following induction of GFP expression during ‘recovery’ growth (after 24 h N starvation). The *insert* is a schematic representation of the experiment. (**B)** Quantification of GFP production over 8 h of ‘recovery’ growth (*insert*) was obtained from the geometric means of GFP fluorescence. Error bars represent sd (*n* = 3).

Since many bacteria adjust their metabolism as an adaptive response to prolonged conditions of nutritional adversity, we considered whether the short lag phase displayed by the ∆*yeaG* mutant strain during ‘recovery’ growth was indicative of an altered metabolic state *ergo* adaptive response between ∆*yeaG* mutant and wild-type strains to sustained N starvation. Accumulation of GFP has been widely used as a reporter for bacterial metabolic status^14^,^15^. Therefore, to investigate how *E. coli* adjust their metabolic state in response to N sustained starvation and to determine if this differs between the wild-type and ∆*yeaG* mutant strains, we assessed the rate of production of green fluorescent protein (GFP) at the single-cell level as a measure for metabolic activity during recovery growth in the presence of the inducer. Bacterial cells were recovered at hourly time points and fluorescence levels were determined by flow cytometry. Results in Fig. 4A,B, show that GFP production in the wild-type strain first became apparent at 3 h, although, strikingly, only in a subset (25%) of the population. By 4 h into ‘recovery’ growth the majority (77%) of the wild-type population contained GFP-producing bacteria and the increase in fluorescence levels continued progressively. However, a small proportion of the population remained non-fluorescent even after 5 h into ‘recovery’ growth. In marked contrast to the wild-type strain, GFP production could be detected in the ∆*yeaG* mutant strain population after only 2 h into ‘recovery’ growth and fluorescence levels increased at a constant rate over time. Strikingly, in the ∆*yeaG* population, the N starvation-induced metabolic heterogeneity clearly evident in the wild-type population was not detected. Overall, results are consistent with the ‘recovery’ growth characteristics of both strains (Fig. 1D), and indicate that the short lag phase observed for the ∆*yeaG* mutant strain during ‘recovery’ growth is likely due to an increased metabolic activity despite the mutant cells having experienced sustained N starvation. Importantly, the results also reveal differences between ∆*yeaG* mutant and wild-type strains at the population level: Whereas the wild-type population displayed heterogeneity in its metabolic activity and contained a small sub-population that appeared to be metabolically inactive, this was clearly not the case in the ∆*yeaG* mutant population.

### The *yeaG*-dependent adaptive response to sustained N starvation involves the transcriptional repression of *mqsR/mqsA* and *dinJ/yafQ* toxin-antitoxin genes

**Figure 5:**
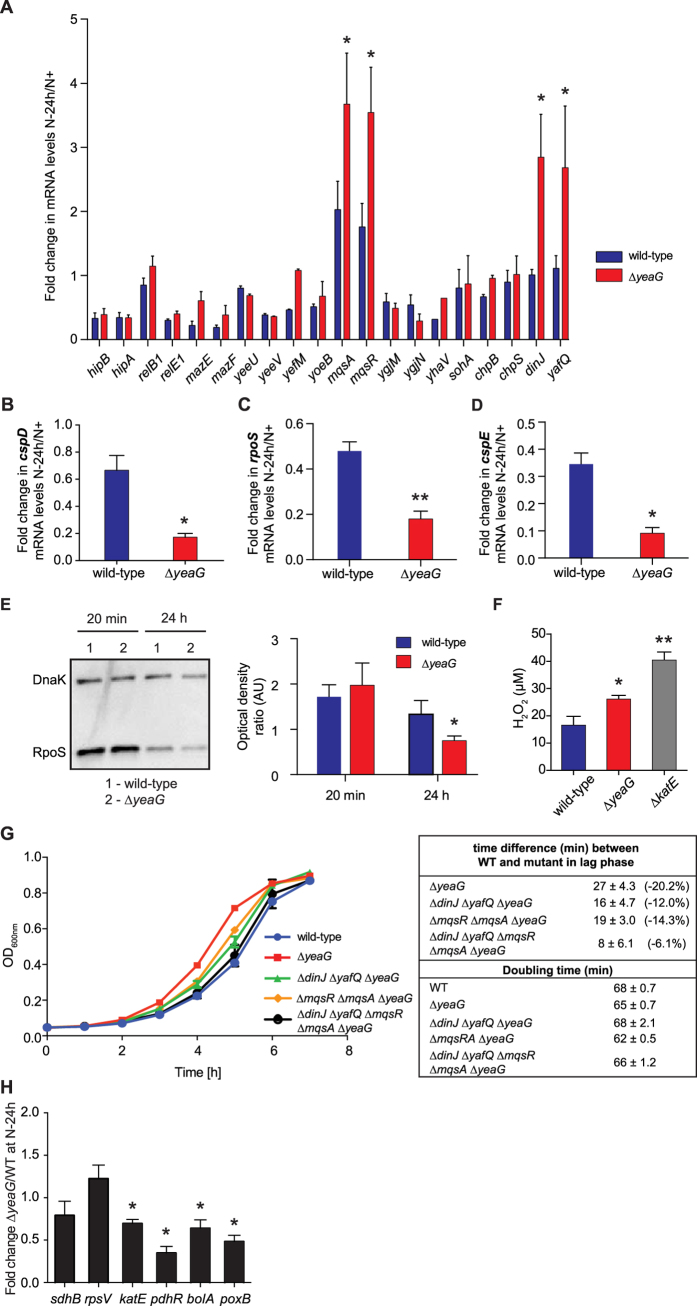
The *yeaG*-dependent adaptive response to sustained N starvation involves the transcriptional repression of *mqsR*/*mqsA* and *dinJ*/*yafQ* toxin-antitoxin genes. (**A**) Expression levels of ten *E. coli* TA gene pairs in wild-type and ∆*yeaG* mutant strain calculated as fold change in mRNA between N+ and N-24 h N-starved conditions and normalised to 16S expression. Error bars represent sd (*n* = 2; except *mqsR*/*mqsA*, where *n* = 4). (**B–D**) Expression levels of *cspD, rpoS* and *cspE* in wild-type and ∆*yeaG* mutant strain calculated as fold change in mRNA between N+ and N-24 h N starved conditions and normalised to 16S expression. Error bars represent sd (*n* = 4). (**E**) Levels of RpoS are reduced in ∆*yeaG* mutant strain following 24 h N starvation. Representative immunoblot of whole-cell extracts of bacterial cells sampled after 20 min and 24 h of N starvation probed with anti-RpoS and anti-DnaK (loading control) antibody. Graph shows quantification of bands by densitometry (*n* = 3), calculated as the ratio in Optical Density between the bands corresponding to RpoS and DnaK. Error bars represent sd (*n* = 4). (**F**) RpoS-dependent catalase activity is impaired in the ∆*yeaG* mutant strain following 24 h of N starvation. Catalase activity was assessed as a function of concentration of H_2_O_2_ remaining in solution after incubation with 24 h N-starved wild-type, ∆*yeaG* or ∆*katE* bacterial lysates. Error bars represent sd (*n* = 3). (**G**) Deletion of *dinJ*/*yafQ* and *mqsR/mqsA* TA modules in the ∆*yeaG* strain leads to complementation of the mutant phenotype, whilst deletion of only one TA modules in the ∆*yeaG* strain leads to only partial complementation. The ‘recovery’ growth curves for wild-type, ∆*yeaG*, ∆*dinJ*∆*yafQ*∆*yeaG, ∆mqsR∆mqsA∆yeaG* and ∆*dinJ*∆*yafQ∆mqsR∆mqsA∆yeaG* mutant strains were obtained by OD_600nm_ readings at hourly time points. (**H.**) Expression levels of *sdhB, rpsV, katE, pdhR, bolA and poxB* at N-24 calculated as fold change in mRNA between the wild-type and ∆*yeaG* mutant strain and normalised to 16S expression. Error bars represent sd (*n *= 3). Statistical analyses for all data-sets were performed by Student’s *t* test (*P < 0.05; **P < 0.01) relative to the wild-type strain.

Toxin-Antitoxin (TA) genes, which are ubiquitous in bacteria, are key effectors that link bacterial growth to the nutritional status of the bacterial cell^16^. Most widely studied TA modules consist of two genes in an operon, which encode for a stable toxin that disrupts essential cellular processes, leading to reversible growth arrest, and a labile antitoxin that forms a tight complex with the toxin and neutralises its effect^17^. The ratio between toxin and antitoxin has important implications in how bacterial cells adapt to stress. During nutritional starvation the antitoxin is degraded, thus generating sufficient free toxin to influence the metabolic status and induce population heterogeneity^17^. Since results in Fig. 4 reveal that *E. coli* adjusts its metabolism in response to sustained N starvation, resulting in a metabolically heterogeneous population, we decided to investigate the relative expression levels of ten known *E. coli* TA gene pairs in ∆*yeaG* and wild-type cells following 24 h N starvation. Results showed that the mRNA levels of the *mqsR/mqsA* TA module to be increased by 2.0 ± 0.9/1.8 ± 0.7 fold in wild-type bacteria following 24 h of N starvation compared to N+ conditions (Fig. 5A). Notably, we detected an even larger increase in mRNA levels corresponding to *mqsR*/*mqsA*, by 3.7-fold ± 1.6/3.5-fold ± 1.4 fold in the ∆*yeaG* mutant strain following 24 h of N starvation compared to N+ conditions (Fig. 5A). Further, in the ∆*yeaG* mutant strain another TA module, *dinJ/yafQ*, was upregulated by 2.8 ± 0.9/2.7 ± 1.3 fold, following 24 h of N starvation compared to N+ conditions. In contrast, *dinJ/yafQ* was not upregulated in the wild-type strain (Fig. 5A). We next focused on the biology of *mqsR/mqsA* and *dinJ/yafQ* to decipher how both of these TA modules could contribute to the properties of the ∆*yeaG* mutant strain: The *mqsR/mqsA* TA module is linked to regulation of the general stress response because it directly represses the transcription of *rpoS*, which encodes for σ^38^, the RNA polymerase associated σ factor that is responsible for executing the general bacterial stress response, and *cspD*, which encodes for the cold-shock protein D, a DNA replication inhibitor^18^,^19^. Consistent with the increased levels of *mqsR/mqsA* mRNA levels in 24 h N-starved ∆*yeaG* mutant compared to wild-type strain (Fig. 5A), mRNA levels of both *rpoS* and *cspD* genes were reduced by 2.8 ± 0.8 fold and 3.92 ± 0.77 fold respectively in 24 h N-starved ∆*yeaG* mutant compared to wild-type strain (Fig. 5B,C). Similarly, DinJ, the toxin component of the *dinJ/yafQ* TA module, has been implicated in reducing σ^38^ levels through directly repressing the transcription of *cspE*, which in turn positively affects σ^38^ translation through stabilisation of *rpoS* mRNA^20^. Consistent with the increased levels of *dinJ*/*yafQ* mRNA in the ∆*yeaG* mutant compared to the wild-type strain following 24 h of N starvation (Fig. 5A), *cspE* mRNA levels were reduced by 4.26 ± 1.82 fold in the ∆*yeaG* mutant compared to the wild-type strain (Fig. 5D). To further corroborate these results, we compared σ^38^ protein levels and activity, which we expected to be reduced in ∆*yeaG* mutant relative to the wild-type strain following 24 h N starvation: As shown in Fig. 5E, σ^38^ levels were 1.7 ± 0.3 fold reduced in the ∆*yeaG* compared to the wild-type strain. Since the transcription of *katG* and *katE*, the genes encoding two catalases capable of converting harmful hydrogen peroxide (H_2_O_2_) into water and oxygen is σ^38^-dependent and thus serves as a surrogate for σ^38^ activity, we compared catalase activity in the wild-type, ∆*yeaG* and ∆*katE* mutant bacteria. Levels of H_2_O_2_ remaining in solution were quantified following incubation with whole-cell lysates from the three strains following 24 h of N starvation. Although the effect was not as accentuated as with the ∆*katE* mutant strain, the catalase activity of the ∆*yeaG* mutant strain was significantly reduced compared to that of the wild-type strain (Fig. 5F), consistent with the reduced levels of σ^38^ in the ∆*yeaG* mutant strain (Fig. 5E). Next, to directly link the property of the ∆*yeaG* mutant strain is mediated by the differences in the levels of expression of *mqsR*/*mqsA* and *dinJ*/*yafQ*, we constructed ∆*dinJ*∆*yafQ*∆*yeaG* and *∆mqsR∆mqsA∆yeaG* triple deletion mutants as well as a ∆*dinJ*∆*yafQ*∆*mqsR*∆*mqsA*∆*yeaG* quintuple deletion mutant. As shown in Fig. 5G, and as expected, the ‘recovery’ growth curve of the ∆*dinJ*∆*yafQ*∆*mqsR∆mqsA*∆*yeaG* mutant strain almost fully resembled that of the wild-type strain, whilst those of the ∆*dinJ*∆*yafQ*∆*yeaG* and *∆mqsR∆mqsA∆yeaG* mutant strains partly resembled that of the mutant strain. Overall, we conclude that the *yeaG*-dependent adaptive response to sustained N starvation is mediated by the transcriptional repression of both *mqsR/mqsA* and *dinJ/yafQ* in *E. coli* cells that experience sustained N starvation. The absence of *yeaG* results in the transcriptional de-repression of *mqsR/mqsA* and *dinJ/yafQ* transcription, which, as a consequence, has a detrimental effect on σ^38^-dependent transcriptional programme in the adaptive response to sustained N starvation. To further substantiate this view, we compared the expression levels of selected genes that are either positively (*rpsV, katE, pdhR, bolA* and *poxB*) or negatively (*sdhB*) regulated by the σ^38^ containing RNA polymerase by qRT-PCR in the ∆*yeaG* mutant and wild-type strains following 24 h of N starvation. Consistent with the results obtained so far, four out of five of the genes (*katE, pdhR, bolA* and *poxB*) that are subjected to positive regulation by σ^38^ showed reduced levels of expression in the ∆*yeaG* mutant strain; the expression level of *rpsV* did not significantly differ in the ∆*yeaG* mutant and wild-type strains following 24 h of N starvation (Fig. 5H). Whereas *sdhB* is negatively regulated by the σ^38^ containing RNA polymerase in the stationary phase of growth in rich medium through promoter occlusion^21^, it seems that reduced σ^38^ levels in the ∆*yeaG* strain does not detectably affect the expression levels of *sdhB* in 24 h N-starved *E. coli*.

### The absence of *yeaG* reduces bacterial cell viability during sustained N starvation

**Figure 6:**
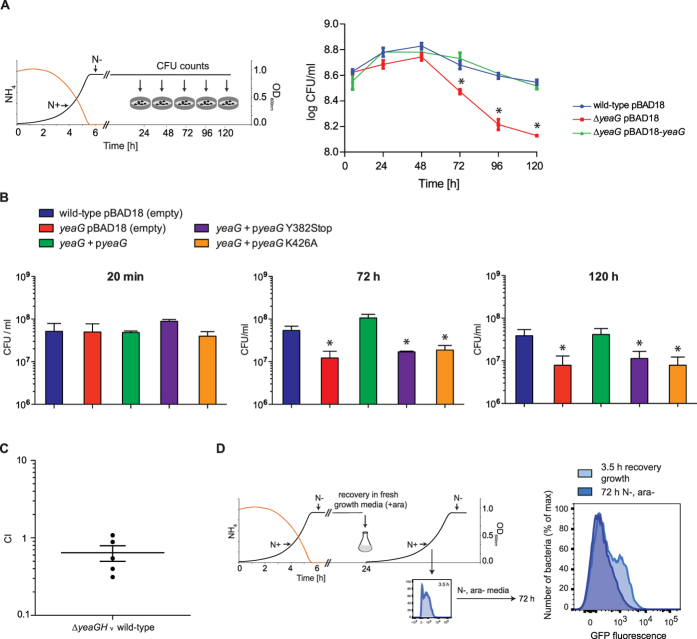
The absence of *yeaG* reduces bacterial cell viability during sustained N starvation. (**A**) The viability of wild-type, ∆*yeaG* mutant and ∆*yeaG* mutant complemented with plasmid-borne YeaG (∆*yeaG* pBAD18-*yeaG*) during sustained N starvation was determined by colony-forming unit (CFU) count over 5 days. Error bars represent s.e.m. (*n *= 3). Statistical analyses were performed by one-way ANOVA (*P < 0.05). (**B**) The viability of wild-type, ∆*yeaG*, ∆*yeaG* complemented with plasmid-borne YeaG (∆*yeaG* pBAD18-*yeaG*) or ∆*yeaG* mutant strain expressing variants of YeaG with deleterious mutations in the eSTK catalytic domain (pBAD18-*yeaG* K426A; pBAD18-*yeaG* Y382Stop) was determined by colony-forming unit (CFU) count after 20 min, 72 h & 120 h in N starvation. Error bars represent sd (*n* = 3). Statistical analyses were performed by one-way ANOVA (*P < 0.05). (**C**) Competitive Index (CI) analysis of *Salmonella* Typhimurium ∆*yeaGH*. C57 BL/6 mice (*n* = 5) were inoculated intragastrically with equal numbers of wild-type and mutant strain. Bacteria were recovered from infected spleens after 5 days post-inoculation and strains distinguished based on antibiotic resistance by replica plating. A CI value of 0.64 (*p* = 0.037), indicating moderate attenuation of the ∆*yeaGH* mutant strain *in vivo*, was obtained by calculating the ratio of wild-type to ∆*yeaGH* bacteria recovered (output), divided by the ratio of wild-type to ∆*yeaGH* mutant bacteria present in the inoculum (input). The scatter plot displays values obtained for individual mice and the mean is indicated. (**D**) Metabolically-inactive bacteria are more likely to withstand long-term stress. Representative histogram of GFP fluorescence shows wild-type population distribution according to metabolic activity at selected time points. The insert diagram shows the levels of GFP fluorescence measured at 3.5 h following induction of GFP expression during ‘recovery’ growth. Bacteria were subsequently transferred to media without inducer or N and GFP fluorescence was assessed again after 24 h in starvation conditions (see text for details).

Since σ^38^-dependent gene expression is central to full adaptation to diverse stress conditions, including N starvation, and thus ultimately to the survival of the bacterial cell, we next investigated how the deletion of *yeaG* impacts bacterial survival following sustained N starvation. Wild-type and ∆*yeaG* mutant bacterial numbers were assessed by plate count of CFU during N starvation over 5 days. As shown in Fig. 6A, following an initial period of little change, bacterial numbers began to drop after 72 h of N starvation for both strains. For the wild-type strain there is a slow but steady decrease in bacterial numbers. However, in comparison, the viability of the ∆*yeaG* mutant strain declines significantly faster. This faster rate of death was not observed in the ∆*yeaG* mutant strain that was complemented with a plasmid-borne wild-type copy of *yeaG,* whose bacterial numbers were comparable to the wild-type strain throughout sustained N starvation (Fig. 6A). However, as expected, a similar decrease in the number of viable bacterial cells was observed after 72 h of N starvation when the ∆*yeaG* mutant strain was complemented with catalytic mutant variants of *yeaG* (Fig. 6B). This observation further corroborates previous results (Fig. 3), which suggests that the catalytic activity of the AAA+ and STK domain of YeaG is important for its function. We thus conclude that absence of *yeaG* impairs the ability of *E. coli* to survive sustained periods of N starvation, most likely because the σ^38^-dependent adaptive response cannot be fully executed. Consistent with a role for YeaG in allowing bacteria to adapt to adverse growth environments, a comparative analysis of virulence carried out with a *S*. Typhimurium ∆*yeaGH* and wild-type strains indicated that mutant bacteria displayed lower proliferation in the spleen (Fig. 6C), thus implying that *yeaG* could be a determinant the overall fitness of *S*. Typhimurium.

### The population of wild-type *E. coli* with increased metabolic activity following sustained N starvation displays impaired viability

The absence of *yeaG* clearly has a detrimental effect on the viability of *E. coli* during sustained period of N starvation (Fig. 6). Results also suggest that, unlike the wild-type strain, the ∆*yeaG* strain exists in a single population with increased metabolic activity following sustained N starvation (Fig. 4). To determine if the differences in population metabolic heterogeneity result in reduced viability of the ∆*yeaG* mutant compared to the wild-type strain following sustained periods of N starvation, we took advantage of the heterogeneous population profile of the wild-type bacterial population during ‘recovery’ growth (Fig. 4). After 3.5 h of ‘recovery’ growth, where metabolic heterogeneity in the wild-type population was evident (Fig. 4), bacteria were transferred to fresh growth media without arabinose (the inducer for GFP expression) and N, thereby stopping both GFP production and bacterial growth. The aim here was to ‘freeze’ the existing population profile that was present at 3.5 h during ‘recovery’ growth. After a further period of 72 h in N starvation, the decrease in CFU in the bacterial populations seen in Fig. 6A was accompanied by the disappearance of the wild-type population displaying increased metabolic activity (i.e. increased GFP production, representing 34% of the total bacteria analysed) (Fig. 6D). Because pre-formed matured GFP molecules are stable for at least 96 h inside live bacterial cells^22^, the loss of bright GFP bacteria can be attributed to bacterial death. We conclude that the population of wild-type *E. coli* with increased metabolic activity following sustained N starvation displays impaired viability. Therefore, the ∆*yeaG* mutant strain is less viable than the wild-type strain, because it, unlike the latter, exists in a single and metabolically active population following sustained N starvation (Figs 4 and 6A).

## Discussion

The operon containing the *yeaG* and *yeaH* genes is highly conserved in *E. coli* and related bacteria. Results from several transcriptomics studies have shown that this operon is expressed in response to a diverse range of stresses. Whilst *yeaH* encodes a protein of little sequence or structural similarity to any known proteins, the product of *yeaG* is an eSTK. The impetus for the present study was the lack of a biological role for *yeaG* in bacterial stress responses. We have now assigned a role for *yeaG* in the adaptation of *E. coli* to sustained N starvation.

**Figure 7:**
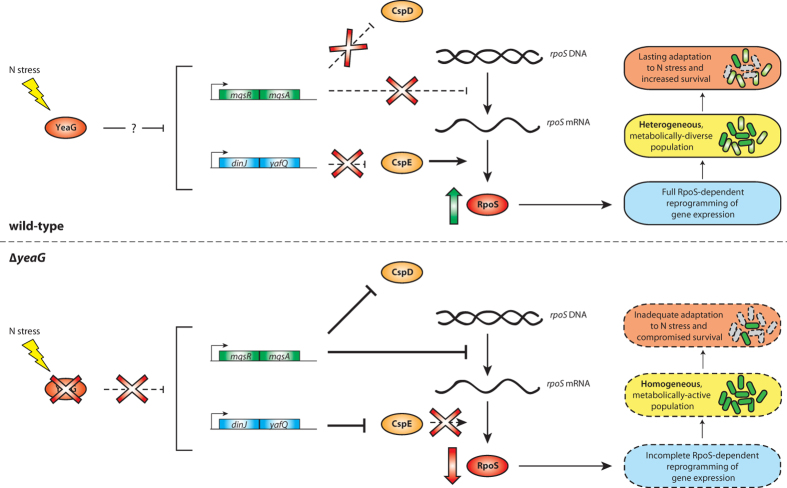
A proposed a role for *yeaG* in ensuring sustained adaptation to sustained N stress in *E. coli* (see text for details).

It is well established that *rpoS* is not expressed in exponentially growing bacteria and the regulation of its expression is tightly controlled at the transcription, translation and protein stability and activity levels as it impacts multiple physiological properties of the cell that affect growth. Therefore, managing transcription and translation of *rpoS* is an essential aspect of the bacterial stress response. Our results suggest that *yeaG* acts upstream of *rpoS* in the regulatory cascade that allows adaptation to sustained N starvation. In the scenario proposed in Fig. 7, in response to sustained N starvation, YeaG represses the transcription of *mqsR/mqsA* and *dinJ*/*yafQ* TA genes. This positively impacts *rpoS* transcription and translation, which as a consequence will result in the execution of the *rpoS*-dependent gene expression programme, needed to fully implement the adaptive response to sustained N stress. Conversely, in the absence of *yeaG*, our results show that the transcription of *mqsR/mqsA* and *dinJ*/*yafQ* TA genes become de-repressed in response to sustained N starvation. This negatively impacts *rpoS* at the transcriptional and translational levels and collectively results in an incomplete execution of the *rpoS*-dependent adaptive response to sustained N stress. Interestingly, a Δ*rpoS* mutant grows poorly compared to the wild-type and ∆*yeaG* strains in the recovery growth experiment (Figure S2), We thus propose that the correct intracellular levels of *rpoS* is important to instigate the appropriate adaptive response to sustained N starvation, whereas the absence of *rpoS* is likely to have significant pleiotropic effects on how *E. coli* adapts to N stress. This observation further underscores the view that *yeaG*, acting upstream of *rpoS*, ensures that intracellular levels of *rpoS* in *E. coli* exposed to sustained N starvation are maintained to allow the appropriate adaptive response to be executed. The mechanistic basis by which the YeaG mediated repression of *mqsR/mqsA* and *dinJ/yafQ* transcription occurs remains elusive and will be the subject of future studies. However, since MqsR, MqsA, DinJ or YafQ are not phosphorylated in a global phosphoproteome analysis of *E. coli* cells from stationary phase batch cultures grown in minimal media^23^, it is unlikely that they serve as substrates for YeaG. It is well established that a variety of regulatory mechanisms (anti-adaptor proteins, small RNAs) contribute to increasing σ^38^ levels (via either increased *rpoS* translation and decreased σ^38^ degradation) in response to suboptimal growth conditions^24^,^25^,^26^,^27^ including nutrient deprivation, and thus we cannot exclude an effect of YeaG on these mechanisms. Nevertheless, we previously reported that NtrC couples the Ntr stress response and the stringent response via the activation of *relA* transcription^1^,^4^. The new results now indicate that the full coupling of these two major bacterial stress responses to N stress adaptation may also involve YeaG. Further, the results also highlight the requirement for the three major *E. coli* σ factors for implementing the full Ntr response: σ^54^ (for the NtrC-mediated activation of Ntr genes, including *yeaG*), σ^70^ (for nitrogen assimilation control protein (Nac)-mediated regulation of Ntr response genes) and σ^38^ (for the general stress response).

By generating phenotypically diverse subpopulations, bacteria employ so called ‘bet-hedging’ strategies to maximise survival by increasing the chance that a small percentage of the population, called persisters, will be better adapted to any sustained adverse change in the growth environment^28^. We have demonstrated that sustained N starvation results in the generation of a metabolically heterogeneous population in a *yeaG*-dependent manner. This seems to clearly confer a physiological advantage by promoting bacterial survival during sustained periods of N starvation. Our results imply that *yeaG* might potentially influence for the formation of persisters during sustained N starvation that can be less susceptible to killing by antibiotics. Notably, and in support of this view, the transcription of *cspD*, a gene required for persister formation^29^, becomes de-repressed during sustained N starvation through the *yeaG*-mediated repression of *mqsR/mqsA* transcription.

In summary, this study provides new insights into mechanisms used by *E. coli* to adapt to sustained N starvation. Further, since *E. coli* is still one of the most widely used systems for the production of recombinant proteins in industrial bioprocessing, managing changes in metabolic performance, such as augmenting the metabolic activity and population uniformity in the ∆*yeaG* strain, could represent a novel route for optimising such processes. This is especially relevant since phenotypic heterogeneity is a major obstacle in the context of improving the robustness of bioprocessing procedures involving bacteria^30^. Further, since the metabolic state clearly influences the susceptibility of bacteria to antibiotics^31^, these findings could potentially be exploited as an innovative way to combat antibiotic-recalcitrant bacteria that cause disease. Importantly, this study has uncovered a novel aspect of the regulatory basis that underpins the Ntr response in *E. coli* and highlights the dynamic and layered complexity of bacterial stress response networks.

## Methods

### Bacterial strains, plasmids and growth conditions

The strains used in this study were derivatives from *Escherichia coli* K-12 or *Salmonella* Typhimurium strain NTCC 12023 and are listed in Table S2 in the supplementary material. The *E. coli* ∆*yeaGH* and *S.* Typhimurium ∆*yeaGH* double mutant strains were constructed using the λ red recombinase method for gene deletion^32^; briefly, a kanamycin resistance cassette was introduced in the bacterial chromosome at the location of the two adjacent genes, thereby replacing them. The *E. coli* ∆*dinJ*∆*yafQ*∆*yeaG* triple mutant strain was obtained by transduction of the knockout *yeaG* allele from ∆*yeaG* (donor strain) into the ∆*dinJ*∆*yafQ* double mutant (recipient strain) using the P1 bacteriophage. Similarly the *E. coli* ∆*dinJ*∆*yafQ∆mqsR∆mqsA*∆*yeaG* quintuple mutant was obtained by transduction of the knockout *mqsRA* allele from ∆*mqsR∆mqsA* (donor strain) in to the triple mutant ∆*dinJ*∆*yafQ*∆*yeaG* (recipient strain) cured of its kanamycin cassette using pCP20, using the P1 bacteriophage. Bacteria were grown at 37 °C, 180 rpm, in Gutnick minimal media (33.8 mM KH_2_PO_4_, 77.5 mM K_2_HPO_4_, 5.74 mM K_2_SO_4_, 0.41 mM MgSO_4_), supplemented with Ho-LE trace elements^33^ and 0.4% (w/v) glucose, and containing either 10 mM NH_4_Cl (for precultures) or 3 mM NH_4_Cl (for ‘pre-starvation’ and ‘recovery’ growth). In all growth assays, bacteria were subcultured in 50 ml of growth media for a starting OD_600_ of 0.05. Where appropriate, growth medium was supplemented with 50 μg/ml ampicillin. For strains carrying complementation (pBAD18) or dual-fluorescence (pFCcGi) plasmids, arabinose was added at 0.1% and 0.2% (w/v), respectively, for induction of gene expression. Plasmids used in this study are listed in Table S2 in the supplementary material. Ammonium concentration in the media was calculated using the Aquaquant ammonium quantification kit (Merck Millipore), according to the manufacturer’s instructions.

### Western Blotting

For immunoblotting, the following commercial antibodies were used: mouse monoclonal anti-RpoS (WP009), at a dilution of 1 : 5 000 (NeoClone), and mouse monoclonal anti-DnaK (8E2/2), at a dilution of 1 : 10 000 (Enzo Life Sciences), in conjunction with the anti-mouse ECL horseradish peroxidase (HRP)-linked secondary antibody, at a dilution of 1 : 10 000, and mouse monoclonal anti-polyHistidine-Peroxidase (A7058), at a dilution of 1 : 5 000 (Sigma-Aldrich).

### Quantitative real-time PCR (qRT-PCR)

RNA samples were obtained from bacteria at specific time points by stabilisation with Qiagen RNA Protect reagent (Qiagen) and extracted using Qiagen RNeasy Mini kit (Qiagen) and PureLink DNase Set (Invitrogen). Purified RNA was stored at −80 °C in nuclease-free water. cDNA was amplified from 100 ng of RNA using the High-Capacity cDNA Reverse Transcription kit (Applied Biosystems). qRT-PCR reactions were as follows: 1 μl cDNA, 5 μl TaqMan Fast Universal PCR Master Mix (Applied Biosystems), 0.5 μl TaqMan probe (Applied Biosystems), in a total volume of 10 μl. Amplification was performed on an Applied Biosystems StepOnePlus Real-Time PCR system using the following conditions: 50 °C (2 min), 95 °C (10 min), followed by 40 cycles of 95 °C (15 s) and 60 °C (1 min). Primer and probe mixtures were custom designed (Custom TaqMan Gene Expression Assays, Applied Biosystems) and sequences are provided in Table S3 in the supplementary material. For analysis of TA mRNA, qRT-PCR reactions were performed using SybrGreen PCR master mix (Applied Biosystems) with 400 ng cDNA and 2 μM specific primers (listed on Table S3). Amplification was done on Rotor-Gene 3000 (Corbett Life Science) with the following cycling parameters: 95 °C (10 min), followed by 45 cycles of 95 °C (10 s), 55 °C (30 s), 68 °C (15 s) and 72 °C (40 s). All real-time analysis was performed in triplicate and quantification of 16S expression served as internal control.

### Measurement of bacterial growth and viability

To establish growth curves, bacterial growth was measured over time by optical density (OD_600nm_). In ‘recovery’ growth, similar numbers of viable cells were confirmed at the starting point. To measure bacterial replication by Fluorescence Dilution, dilution of GFP fluorescence was assessed in ‘recovery’ growth, in the absence of arabinose, with pre-induced strains carrying pFCcGi. At selected times, bacteria were fixed using 2% (w/v) paraformaldehyde in PBS for 10 min at room temperature, resuspended in PBS solution and kept at 4 °C until analysis by flow cytometry. Bacterial replication was measured as the fold change in the geometric mean values of GFP fluorescence intensity between a given time point and 0 h. Number of viable cells was determined by colony forming unit (CFU) ml^−1^ of bacteria at selected time points, by plating serial dilutions onto LB agar. Bacterial growth was also measured as the fold change in CFU ml^−1^ between selected time points and 0 h.

### Flow cytometry

For each sample, 10,000 bacterial events were acquired on a LSR Fortessa flow cytometer (BD) using FACSDiva software. GFP and mCherry fluorescence intensities were detected at 525/50 nm and 610/20 nm, respectively. Data analysis was done using the FlowJo software version 10 (TreeStar). To analyse GFP fluorescence, bacteria were previously gated on mCherry-positive signal.

### Catalase Activity

Catalase activity of bacterial cells was measured using Catalase Assay kit (Sigma-Aldrich), according to the manufacturer’s instructions. Bacteria were pelleted from 1 ml aliquot of culture, washed with Assay Buffer and lysed by sonication, prior to colorimetric assay for calculation of hydrogen peroxide substrate remaining in solution.

### Competitive infections

All animal experiments were performed in accordance with the Animals Scientific Procedures Act 1986 and were approved by the Imperial College London Ethical Review Committee. Infection studies were carried out with 6–8 week old female C57BL/6mice (Charles River). Groups of 5 mice were inoculated intragastrically with 0.2 ml of bacteria resuspended in PBS solution. The bacteria inoculum was 5 × 10^8^ CFU of combined wild-type and mutant strains (2.5 × 10^8^ of each strain, at a ratio of 1:1). Bacterial input was quantified by plating serial dilutions of the inoculum and strains were differentiated based on antibiotic resistance. Mice were sacrificed after 5 days and dilution series of spleen lysates were plated for CFU count (output) and strains were differentiated based on antibiotic resistance. Values for Competitive Index (CI) were calculated as the ratio of mutant to wild-type in the output divided by that of the input.

### Bioinformatics

Alignments were performed using the Molecular Evolutionary Genetics Analysis (MEGA) software version 6^34^. Phylogenetic dendrogram was obtained from multiple sequence alignments using the maximum likelihood method. The full amino acid sequence of *E. coli* YeaG was used in the analysis.

### Statistical analysis

Unless specified otherwise, data is represented as the mean average of 3 independent experiments or more and variation is shown by standard error of mean (s.e.m.). Statistically significant relationships were determined using Student’s *t*-test or one-way analysis of variance (ANOVA), with a probability (*p*) value < 0.05 being the statistical significance considered.

## Additional Information

**How to cite this article**: Figueira, R. *et al.* Adaptation to sustained nitrogen starvation by *Escherichia coli* requires the eukaryote-like serine/threonine kinase YeaG. *Sci. Rep.* **5**, 17524; doi: 10.1038/srep17524 (2015).

## Supplementary Material

Supplementary Information
