# Supplementary material for: Adaptation to sustained nitrogen starvation by Escherichia coli requires the eukaryote-like serine/threonine kinase YeaG
Source: Sci Rep. 2015 Dec 1;5:17524. doi: 10.1038/srep17524 (PMC4664914; doi:10.1038/srep17524)
Supplement: Supplementary Information [file srep17524-s1.pdf]

Supplementary Information

Adaptation to sustained nitrogen starvation by *Escherichia coli* requires the eukaryote-like serine/threonine kinase YeaG

Rita Figueira, Daniel R. Brown, Delfim Ferreira, Matthew J. G. Eldridge, Lynn Burchell, Zhensheng Pan, Sophie Helaine and Sivaramesh Wigneshweraraj*

MRC Centre for Molecular Microbiology and Infection, Imperial College London, UK

*To whom correspondence should be addressed: Sivaramesh Wigneshweraraj MRC Centre for Molecular Microbiology and Infection, Imperial College London, SW7 2AZ, United Kingdom, Tel: +44 (0)207 594 1867, Fax: +44 (0)207 594 3055, E-mail: s.r.wig@imperial.ac.uk

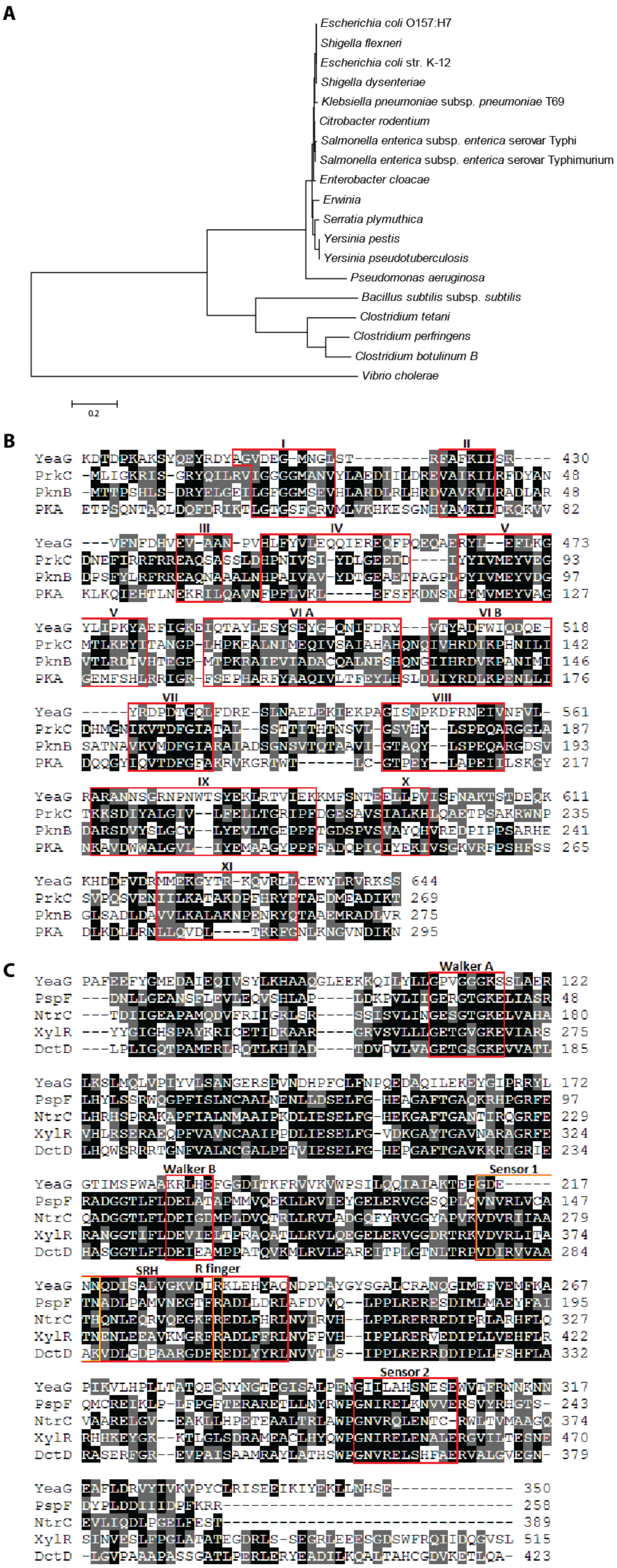
A
Escherichia coli O157:H7
Shigella flexneri
Escherichia coli str. K-12
Shigella dysenteriae
Klebsiella pneumoniae subsp. pneumoniae T69
Citrobacter rodentium
Salmonella enterica subsp. enterica serovar Typhi
Salmonella enterica subsp. enterica serovar Typhimurium
Enterobacter cloacae
Erwinia
Serratia plymuthica
Yersinia pestis
Yersinia pseudotuberculosis
Pseudomonas aeruginosa
Bacillus subtilis subsp. subtilis
Clostridium tetani
Clostridium perfringens
Clostridium botulinum B
Vibrio cholerae
0.2
B
YeaG
PrkC
PknB
PKA
I
II
III
IV
V
VI A
VI B
VII
VIII
IX
X
XI
C
YeaG
PspF
NtrC
XylR
DctD
Walker A
Walker B
Sensor 1
SRH
R finger
Sensor 2

FIGURE S1. YeaG is highly conserved within bacterial species and displays marked similarity to known eukaryotic-like serine-threonine kinases (eSTK) and ATPase Associated with diverse cellular Activities (AAA+) proteins. A. Phylogenetic dendrogram displaying the similarity between YeaG from *E. coli* MG1655 and other bacterial species. The rooted maximum likelihood tree was constructed using the MEGA 6 software. **B.** Alignment of the C-terminal eSTK domain from YeaG of *E. coli* MG1655 with well-characterised eSTK proteins: PrkC from *Bacillus subtilis* (1) and PnkB from *Mycobacterium tuberculosis* (2,3), as well as the eukaryotic PKA from *Mus musculus* (3). Kinase subdomains (I to XI) are highlighted and sequences displaying 100% conservation are shaded in black. **C.** Alignment of the N-terminal AAA+ domain from YeaG of *E. coli* MG1655 with well-characterised ATPases: PspF and NtrC from *E. coli*, XylR from *Pseudomonas putida* and DactD from *Sinorhizobium meliloti* (4). Conserved motifs are highlighted and sequences displaying 100% conservation are shaded in black.

1. Madec, E., Stensballe, A., Kjellstrom, S., Cladiere, L., Obuchowski, M., Jensen, O. N., and Seror, S. J. (2003) Mass spectrometry and site-directed mutagenesis identify several autophosphorylated residues required for the activity of PrkC, a Ser/Thr kinase from Bacillus subtilis. *Journal of molecular biology* **330**, 459-472
2. Boitel, B., Ortiz-Lombardia, M., Duran, R., Pompeo, F., Cole, S. T., Cervenansky, C., and Alzari, P. M. (2003) PknB kinase activity is regulated by phosphorylation in two Thr residues and dephosphorylation by PstP, the cognate phospho-Ser/Thr phosphatase, in *Mycobacterium tuberculosis*. *Molecular microbiology* **49**, 1493-1508
3. Pereira, S. F., Goss, L., and Dworkin, J. (2011) Eukaryote-like serine/threonine kinases and phosphatases in bacteria. *Microbiology and molecular biology reviews : MMBR* **75**, 192-212
4. Zhang, X., Chaney, M., Wigneshweraraj, S. R., Schumacher, J., Bordes, P., Cannon, W., and Buck, M. (2002) Mechanochemical ATPases and transcriptional activation. *Molecular microbiology* **45**, 895-903

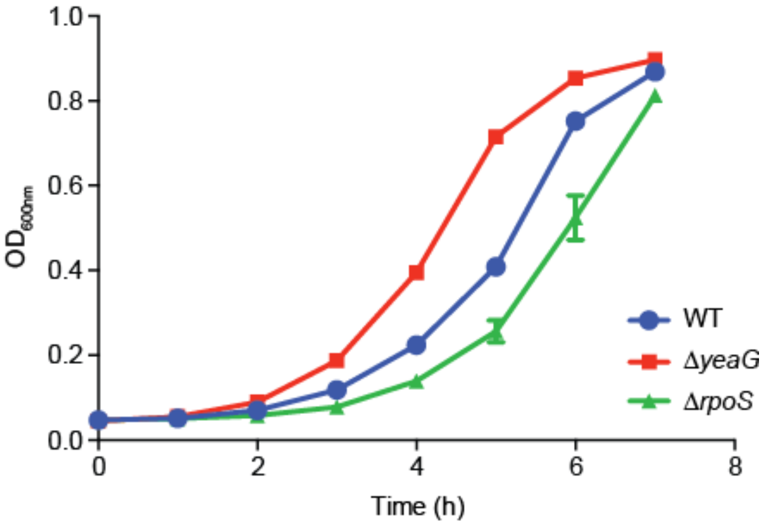

FIGURE S2. The Δ*rpoS* mutant has a longer lag phase during "recovery" growth following sustained N starvation. The 'recovery' growth curves were obtained by determining the OD_{600nm} readings at half-hourly time points.

Table S1. Growth parameters of Δ*yeaH* and Δ*yeaGH* bacteria in 'recovery' growth following 20 min, 12 h, 18 h and 24 h in N starvation. Lag phase defined as the period up to and including OD_{600} = 0.1. The Δ*yeaG* percentage of wild-type lag phase is indicated in brackets. The doubling time was determined as the slope of logarithmic growth function during exponential phase.

Δtime (min) between WT and mutant strains in lag phase (OD_{600nm} = 0.1)		
	Δ*yeaH*	Δ*yeaGH*
20 min (N-)	0.0 ± 2.4 (0.0%)	0.0 ± 1.8 (0.0%)
12h (N-)	21 ± 1.2 (10.0%)	21 ± 0.6 (10%)
18h (N-)	30 ± 1.2 (13.4 %)	28 ± 0.6 (12.5%)
24h (N-)	38 ± 1.8 (15.9%)	39 ± (16.3%)

Strain	time (N-)	Doubling time (min)
Δ*yeaH*	20 min	67 ± 0.2
	12 h	66 ± 2.4
	18 h	67 ± 0.6
	24 h	68 ± 1.2
Δ*yeaGH*	20 min	66 ± 1.2
	12 h	67 ± 0.6
	18 h	67 ± 0.6
	24 h	67 ± 0.6

Table S2. List of strains and plasmids used in this study
Abbreviations: Amp, ampicillin; Km, kanamycin

Strains

Name	Description	Source or Reference
wild-type	*E. coli* str. K-12 substr. MG1655 [F-, λ⁻, Δ(*araD-araB*)*567*, *rph-1*] (BW25113)	*E. coli* Genetic Stock Center
Δ*yeaG*	*E. coli* str. K-12 substr. MG1655 Δ*yeaG780::km* (JW1772-1)	*E. coli* Genetic Stock Center
Δ*yeaH*	*E. coli* str. K-12 substr. MG1655 Δ*yeaH781::km* (JW1773-1)	*E. coli* Genetic Stock Center
Δ*yeaGH*	*E. coli* str. K-12 substr. MG1655 Δ*yeaGH::km*	This study
Δ*rpoS*	*E. coli* str. K-12 substr. MG1655 Δ*rpoS746::km* (JW5437-1)	*E. coli* Genetic Stock Center
Δ*glnG*	*E. coli* str. K-12 substr. MG1655 Δ*glnG730::km* (JW3839-2)	*E. coli* Genetic Stock Center
Δ*katE*	*E. coli* str. K-12 substr. MG1655 Δ*katE731::km* (JW1721-1)	*E. coli* Genetic Stock Center
Δ*dinJ/yafQ*	*E. coli* str. K-12 substr. MG1655 Δ*dinJ/yafQ::km*	Gift from K. Gerdes
Δ*dinJ/yafQ/yeaG*	*E. coli* str. K-12 substr. MG1655 Δ*yeaG* Δ*dinJ/yafQ::km*	This study
S. Typhimurium Δ*yeaGH*	*S. enterica* subsp. *enterica* serovar Typhimurium Δ*yeaGH::km*	This study

Plasmids

Name	Description	Source or Reference
pFCcGi	*rpsM::mCherry* and P_{BAD}*::gfpmut3a* promoter fusions in pFPV25.1 (Amp^R)	(1)
pBAD18-*yeaG*	pBAD18 expressing 6xHis-*yeaG* under an arabinose-inducible promoter (*araC*) (Amp^R)	This study
pBAD18-*yeaG* K116A	pBAD18-*yeaG* containing a K116A point mutation	This study
pBAD18-*yeaG* R232A	pBAD18-*yeaG* containing a R232A point mutation	This study
pBAD18-*yeaG* Y382Stop	pBAD18-*yeaG* containing a Y382 point mutation to a stop codon	This study

pBAD18-*yeaG* K426A	pBAD18-*yeaG* containing a K426A point mutation	This study

1. Figueira, R., Watson, K. G., Holden, D. W., and Helaine, S. (2013) Identification of salmonella pathogenicity island-2 type III secretion system effectors involved in intramacrophage replication of S. enterica serovar typhimurium: implications for rational vaccine design. *mBio* **4**, e00065

Table S3. List of primers used in this study

Name	5' to 3' nucleotide sequence
AAA K116A FW	GTGGGTGGGGGTGCATCATCGCTTGCTG
AAA K116A R	CAGCAAGCGATGATGCACCCCCACCCAC
AAA R232A FW	GGTTGGGAAAGTCGATATTGCTAAACTCGAACACTACG
AAA R232A R	CGTAGTGTTCGAGTTTAGCAATATCGACTTTCCCAACC
STK Y382stop FW	CCAGAAAACTCCAGCATTTAGTCAAAGATGCGGGTTTATG
STK Y382stop R	CATAAACCCGCATCTTTGACTAAATGCTGGAGTTTTCTGG
STK K426A FW	CGTTTTGCGTTTGCGATCCTCTCCCGC
STK K426A R	GCGGGAGAGGATCGCAAACGCAAAACG
hipB FW	CTATAGCCCAACGCAATTGG
hipB R	TTTTTCGCGTCGCATAGCGT
hipA FW	TTGAATGTTCCGGACGCAGA
hipA R	AAAAAAGCCATGATCCGCGC
relB FW	CCCGAATCGATGAAGATCTG
relB R	AACATCAATGCCAGCTTCGC
relE FW	GGGATATTGAATACTCGGGA
relE R	AAATCAGGATCCAGTCCGGT
mazE FW	TAGCGTAAAGCGTTGGGGAA
mazE R	ATTCTCGTGGAGGTTTTCCG
mazF FW	AAAAGGTAGCGAGCAAGCTG
mazF R	CCGCCAGGCGATACTTTTTA
yeeU FW	AACCGGTTGCATTACCTTGC
yeeU R	TCACAACTGCTGAGGGTATC
yeeV FW	ATCTGGCAGATACTGCTGTC
yeeV R	TTAACTGAGAGCGGGTACAG
yefM FW	CAATTAGCTACAGCGAAGCG
yefM R	TTGAGTCCATCAATCTCCGG

yoeB FW	TCTGGTCTGAGGAATCATGG
yoeB R	AGACGGTGCTCCTCTGTAAT
mqsA FW	CCTTCCGTGGACGAAAAACA
mqsA R	CGCTTGCCTCTTTTTGGGTA
mqsR FW	CGCACACCACATACACGTTT
mqsR R	AAACCTGGCCTGTAACAAGC
ygjM FW	CGGGTATTCAGAACGAGGAA
ygjM R	CATAAGGGTACGAATCACGG
ygjN FW	TAAAACGGAGTTGGTGGCTC
ygjN R	CTTAGTACGATGAACAGCGG
yhaV FW	GACGCTTTAGTTGCCGAAGT
yhaV R	CCAGCACCAAATTTTACCCG
sohA FW	CCACTGAATCAAAGGTCACG
sohA R	GGACGAGTTTTTTGCGGGTT
chpB FW	GTGAATTTGAACGGGGAGAC
chpB R	CATCGCCTTCTTCGCAATGT
chpS FW	AAAAGATGGGGGAACAGTGC
chpS R	ACATCCTGCTCGCTAAGTTC
dinJ FW	CCTGCGTATTGACGATGAAC
dinJ R	CTGGCTTAGGATTACGAAGC
yafQ FW	TTCTGGATTTTGACGAGCGG
yafQ R	TGGTATACAAGGCGATAGCC
16S FW	CATAACGTCGCAAGACC
16S R	GGTCATCCTCTCAGACCAG
